# PCSK9 regulates the efficacy of immune checkpoint therapy in lung cancer

**DOI:** 10.3389/fimmu.2023.1142428

**Published:** 2023-03-21

**Authors:** Xiang Gao, Ling Yi, Chang Jiang, Shuping Li, Xiaojue Wang, Bin Yang, Weiying Li, Nanying Che, Jinghui Wang, Hongtao Zhang, Shucai Zhang

**Affiliations:** ^1^ Cancer Research Center, Beijing Tuberculosis and Thoracic Tumor Research Institute/Beijing Chest Hospital, Capital Medical University, Beijing, China; ^2^ Department of Medical Oncology, Beijing Tuberculosis and Thoracic Tumor Research Institute/Beijing Chest Hospital, Capital Medical University, Beijing, China; ^3^ Department of Thoracic Oncology, Jiangxi Cancer Hospital, Nanchang, China; ^4^ Department of Cardiology, Beijing Tuberculosis and Thoracic Tumor Research Institute/Beijing Chest Hospital, Capital Medical University, Beijing, China; ^5^ Department of Pathology, Beijing Tuberculosis and Thoracic Tumor Research Institute/Beijing Chest Hospital, Capital Medical University, Beijing, China

**Keywords:** proprotein convertase subtilisin/kexin type 9, PCSK9, immunohistochemical markers, immunotherapy, advanced non-small cell lung cancer, anti-CD137 agonist, immune infiltration

## Abstract

Proprotein convertase subtilisin/kexin type 9 (PCSK9) secreted by tumors was reported as a deleterious factor that led to the reduction of lymphocyte infiltration and the poorer efficacy of ICIs *in vivo*. This study aimed to explore whether PCSK9 expression in tumor tissue could predict the response of advanced non-small cell lung cancer (NSCLC) to anti-PD-1 immunotherapy and the synergistic antitumor effect of the combination of the PCSK9 inhibitor with the anti-CD137 agonist. One hundred fifteen advanced NSCLC patients who received anti-PD-1 immunotherapy were retrospectively studied with PCSK9 expression in baseline NSCLC tissues detected by immunohistochemistry (IHC). The mPFS of the PCSK9^lo^ group was significantly longer than that of the PCSK9^hi^ group [8.1 *vs*. 3.6 months, hazard ratio (HR): 3.450; 95% confidence interval (CI), 2.166-5.496]. A higher objective response rate (ORR) and a higher disease control rate (DCR) were observed in the PCSK9^lo^ group than in the PCSK9^hi^ group (54.4% *vs*. 34.5%, 94.7% *vs*. 65.5%). Reduction and marginal distribution of CD8^+^ T cells were observed in PCSK9^hi^ NSCLC tissues. Tumor growth was retarded by the PCSK9 inhibitor and the anti-CD137 agonist alone in the Lewis lung carcinoma (LLC) mice model and further retarded by the PCSK9 inhibitor in combination with the CD137 agonist with long-term survival of the host mice with noticeable increases of CD8^+^ and GzmB^+^ CD8^+^ T cells and reduction of Tregs. Together, these results suggested that high PCSK9 expression in baseline tumor tissue was a deleterious factor for the efficacy of anti-PD-1 immunotherapy in advanced NSCLC patients. The PCSK9 inhibitor in combination with the anti-CD137 agonist could not only enhance the recruitment of CD8^+^ and GzmB^+^ CD8^+^ T cells but also deplete Tregs, which may be a novel therapeutic strategy for future research and clinical practice.

## Introduction

Treatment with immune checkpoint inhibitors (ICIs) has revolutionized the field of cancer therapy as a therapeutic strategy to improve the prognosis of several solid cancers. However, unlike targeted therapy, there are no perfect biomarkers predicting the efficacy of ICIs and the prognosis of patients. The disappointing response rate also restricted current immunotherapies. Antitumor immunity is influenced by various factors, and it is of critical importance to identify novel and effective therapeutic biomarkers.

Proprotein convertase subtilisin/kexin type 9 (PCSK9), mainly synthesized by hepatocytes and one of the key enzymes in lipid transport, was correlated with an expanded risk for hypercholesterolemia and coronary artery disease (1). It was also identified as a potential factor in the apoptosis, metastasis, and invasion of tumor cells in several cancer types (2–5). From a standpoint of cell biology, the study of Xu et al. (6) reported that PCSK9 small interfering (si)RNA might exert its antitumor activity by inducing mitochondrial dysfunction and ERS-associated cell death in A549 cells. From a standpoint of immunity, Liu et al. (7) demonstrated that PCSK9 could affect the infiltration of lymphocytes, particularly CD8^+^ T cells, in tumor tissues by interfering with the major histocompatibility complex class I (MHC I) recycling pathway in the tumor cell membrane. In lung cancer, a pilot study has shown that serum PCSK9 levels at the second nivolumab cycle could predict overall survival (OS) in elderly patients with non-small cell lung cancer (NSCLC): low levels of circulating PCSK9 predicted better OS in elderly patients with advanced, pretreated NSCLC (8). Furthermore, it was reported that low baseline plasma PCSK9 level was associated with good clinical outcomes of ICIs in advanced NSCLC (9). The PCSK9 protein distributed in tumor tissue may have a more direct impact on the efficacy of ICIs (7), but no research was conducted discussing the association between efficacy and PCSK9 expression in tumor tissue in advanced NSCLC.

Based on this background, we retrospectively studied the clinical data of a cohort of advanced NSCLC patients who received anti-PD-1 immunotherapy and detected PCSK9 expression by immunohistochemistry (IHC) in baseline NSCLC tissues collected before treatment with ICIs, to identify whether PCSK9 expression in NSCLC tissues was associated with more or less response to anti-PD-1 immunotherapy. Next, multiplexed IHC was used to explore the density and spatial distribution of CD8^+^ T cells with tumor cells in NSCLC tissues with different PCSK9 expression levels. In parallel, considering the process of T-cell activation and the effect of PCSK9 inhibition and CD137 agonist on it, a series of experiments using immunocompetent syngeneic mice were conducted to explore the synergistic antitumor effect of PCSK9 inhibition with CD137 costimulation.

## Materials and methods

### Patients and tissue specimens

This study was approved by the Clinical Research Ethics Committee of Beijing Chest Hospital, Capital Medical University, Beijing, China (YJS-2021-020). Patients enrolled in this retrospective study received anti-PD-1 immunotherapy from February 2016 to September 2021, and tissue specimens were obtained through core needle biopsies under CT guidance or bronchoscopy before immunotherapy. The specimens were routinely paraffin wax-embedded and diagnosed with lung adenocarcinoma or squamous cell carcinoma by immunohistochemistry (hematoxylin–eosin staining, TTF-1, napsin A, CK5/6, and p63). PD-L1 expression (tumor proportion score, TPS) was determined using the Dako PD-L1 IHC 22C3 pharmDx assay (PD-L1 negative: TPS < 1%; PD-L1 low: TPS < 50%; PD-L1 high: TPS ≥ 50%). All the patients (ages 18-85 years) were confirmed to be at stage IIIB–IV and to have an Eastern Cooperative Oncology Group performance status (ECOG PS) of 0-2 with clinical imaging of baseline and follow-up time points. Patients with multiple tumor types and autoimmune disease or who discontinued treatment were excluded.

### Efficacy evaluation

A CT screen was used to monitor the efficacy of immunotherapy during treatment per 2 cycles. These imaging data were reviewed according to RECIST version 1.1 by two independent senior clinical oncologists blinded to the histopathologic results. Progression-free survival (PFS) was defined as the time from the date of initiation of systemic immunotherapy to the date of first documented radiologically confirmed progressive disease or death of any cause. OS was defined as the time from the date of initiation of systemic immunotherapy to the date of death of any cause. Objective response rate (ORR) was defined as the proportion of patients with complete response and partial response, and disease control rate (DCR) was defined as the proportion of patients with complete response, partial response, and stable disease. The patients were followed up until 30 April 2022.

### Immunohistochemistry

Histological sections were deparaffinized and subjected to antigen retrieval with citric acid antigen repair buffer, pH 6.0 (E673002, Sangon Biotech, Shanghai, China). Sections were incubated with rabbit anti-PCSK9 antibody (55206-1-AP, 1:2,000, Proteintech, Chicago, IL, USA) as the primary antibody and with polyclonal anti-rabbit IgG (PV-9001, ZSGB-BIO, Beijing, China) as the secondary antibody. Immunocomplexes were detected using 3,3′-diaminobenzidine (DAB; ZLI-9018, ZSGB-BIO, Beijing, China). All images were acquired using the same microscope and camera set with a visible-light microscope (×200 magnification) of DS-Ri1 (Nikon, Japan). The intensity of positive staining in the cytoplasm of tumor cells was measured by mean integrated optical density (mean IOD) calculated using Image-Pro Plus 6.0 software (Media Cybernetics, Silver Spring, USA): mean IOD = IOD/area of the tumor section. X-tile (10) was used to find a proper cutoff on mean IOD.

### Multiplexed immunofluorescent staining

Please refer to reference (11) for further details of the method and the antibodies used.

#### Mice model and treatment

All animal experiments were approved by the Animal Use and Care Committee of Beijing Chest Hospital (2021-003). Eight-week-old syngeneic female C57BL/6J mice, purchased from Beijing Vital River Laboratory Animal Technology Co. Ltd., were inoculated subcutaneously with Lewis lung carcinoma (LLC) cells (1.0 × 10^6^ per mouse). Five micrograms of anti-PCSK9 monoclonal antibody (evolocumab, Amgen, CA, USA) was intratumorally injected on days 5, 7, 9, and 11 in the PCSK9i group and the doublet group. Three micrograms of anti-CD137 agonist (a rat anti-mouse CD137 agonist, 1D8, US patent #7,754,209 B2, SEQ ID Nos. 101 and 103, mIgG2a isotype) was intratumorally injected on days 9, 11, and 13 in the aCD137 group and the doublet group. Tumor size was measured using a caliper and calculated using the formula—volume = (length)(width)^2^/2—every 2 days afterward. Body weights were also monitored every 2 days. The endpoint was defined as the time in which a progressively growing tumor reached 15 mm in its longest dimension or 2,000 mm^3^ in diameter. Mice were also euthanized when they experienced open skin lesions, were dead, or failed to thrive.

### Analysis of T-cell subsets

Tumors and spleens were harvested on day 21 after inoculation. Single-cell suspensions were prepared by cutting fresh tumor tissues and spleens into small pieces (2-4 mm) using a tumor dissociation kit (130-096-730; mouse, Miltenyi Biotec, Germany) and gentleMACS device according to the manufacturers’ instructions. The single-cell suspensions were washed and erythrocytes (RBCs) were lysed for 3 min. The obtained single-cell suspensions were washed, minced on a 70-μm nylon mesh cell strainer, and incubated for 10 min with anti-mouse CD16/32 (156603, BioLegend, CA, USA) and for 25 min with a set of antibodies, including BV510-anti-CD45 (103188, BioLegend, CA, USA), PerCP/Cyanine5.5-anti-CD3ϵ (100328, BioLegend, CA, USA), FITC-anti-CD4 (100509, BioLegend, CA, USA), APC/FireTN750-anti-CD8a (100766, BioLegend, CA, USA), and BV421-anti-CD137 (740033, BD Biosciences, NJ, USA). After being stained for 10 min with Fixable Viability Stain 440UV (BD Biosciences, NJ, USA), fixed for 50 min with Transcription Factor Buffer (562574, BD Biosciences, NJ, USA) at 4°C, and washed by 1X Perm/Wash buffer (BD Biosciences, NJ, USA), the stained cells were incubated for 50 min with Alexa Flour 647 anti-mouse FOXP3 (126408, BioLegend, CA, USA) and PE anti-human/mouse Granzyme B Recombinant (396406, BioLegend, CA, USA) at 4°C and then washed twice prior to analysis with an LSRFortessa flow cytometer (BD Biosciences, NJ, USA).

### Analysis of serum ALT, TG, TC, and LDL-C levels by ELISA

The plasma of mice was harvested on day 21 after inoculation and converted to serum at 4°C for clotting, and the clot was removed by low-speed centrifugation. Alanine aminotransferase (ALT/GPT) activity and the levels of triglyceride (TG), total cholesterol (T-CHO), and low-density lipoprotein cholesterol (LDL-C) were measured with an assay kit (Jiancheng Bioengineering Institute, Nanjing, China) according to the kit’s manual.

### Statistical analysis

Data analyses were performed using GraphPad Prism 8.0 and SPSS 22.0. The Mann–Whitney test was used to compare non-normally distributed data, and the unpaired *t*-test with Welch’s correction was used to compare normally distributed data with unequal variances between the two groups. Distributions of clinicopathological characteristics were compared *via* the chi-square test or Fisher’s exact test. Survival outcomes were evaluated with the Kaplan–Meier survival estimates, log-rank tests, and univariate and multivariate Cox proportional hazards analyses. Two-way ANOVA was used for multiple comparisons in tumor growth delay experiments and body weight changes. *P*-value <0.05 was considered significant.

## Results

### Association of baseline levels of PCSK9 in tumor tissue with clinicopathologic features in advanced NSCLC patients

**Figure 1:**
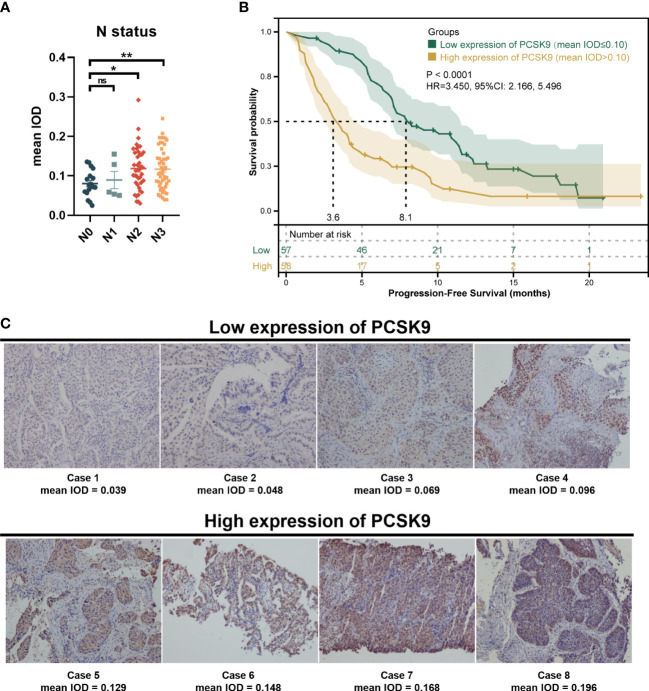
Immunohistochemistry of proprotein convertase subtilisin/kexin type 9 (PCSK9) in advanced non-small cell lung cancer (NSCLC) tissues. **(A)** PCSK9 expression in NSCLC tissues was associated with the N status of advanced NSCLC patients. **(B)** Kaplan–Meier estimate for PFS of the PCSK9^hi^ and PCSK9^lo^ groups by a proper cutoff of mean IOD of PCSK9 expression. The data cutoff was 30 April 2022. **(C)** Immunohistochemistry staining for PCSK9 of NSCLC tissues from advanced NSCLC patients before receiving anti-PD-1 immunotherapy. *ns, No significant difference, *P < 0.05, **P < 0.01.

A total of 115 patients met the inclusion criteria and were included in this retrospective analysis. The expression of PCSK9 measured by immunohistochemistry was different in N status, while it was not associated with other clinicopathologic features (*P* > 0.05) (**Supplementary Figure 1**; **Figure 1A**). The median PFS was 8.1 months in the low PCSK9 group (*n* = 57) which was longer than that of 3.6 months in the high PCSK9 group (*n* = 58) (*P* < 0.0001) (**Figure 1B**). The expression level of PCSK9 in different NSCLC patient tissues varied greatly (**Figure 1C**). There were no statistically significant differences between the two groups for all the clinicopathological characteristics and other factors, including immunotherapy protocols and the number of treatment lines (**Table 1**).

**Table 1:** Clinicopathological characteristics and factors of advanced NSCLC patients in the PCSK9^lo^ and PCSK9^hi^ groups.

Factors	PCSK9^lo^ (*n* = 57)	PCSK9^hi^ (*n* = 58)	*P*
Age, years (%)			0.301
≤65	33 (57.9%)	28 (48.3%)	
>65	24 (42.1%)	30 (51.7%)	
Median (range)	63 (47-81)	66 (34-80)	
Gender, no. (%)			0.860
Male	45 (78.9%)	45 (77.6%)	
Female	12 (21.1%)	13 (22.4%)	
Smoking status, no. (%)			0.892
Never	18 (31.6%)	19 (32.8%)	
Ever	39 (68.4%)	39 (67.2%)	
ECOG performance status, no. (%)			0.717^a^
0–1	54 (94.7%)	53 (91.4%)	
2	3 (5.3%)	5 (8.6%)	
Stage at entry, no. (%)			0.497
IIIB/IIIC	9 (15.8%)	12 (20.7%)	
IV	48 (84.2%)	46 (79.3%)	
Tumor histology, no. (%)			0.506
Squamous cell carcinoma	24 (42.1%)	28 (48.3%)	
Adenocarcinoma	33 (57.9%)	30 (51.7%)	
Mutation, no. (%)			0.823
Negative/*KRAS*	49 (76.0%)	49 (84.5%)	
*EGFR*	8 (14.0%)	9 (15.5%)	
PD-L1 expression, no. (%)			0.878
TPS < 1%	20 (35.1%)	23 (39.7%)	
1% ≤ TPS < 50%	14 (24.6%)	13 (22.4%)	
TPS ≥ 50%	23 (40.3%)	22 (37.9%)	
Remote metastases, no. (%)			0.299
No	23 (24.6%)	29 (50.0%)	
Yes	34 (59.6%)	29 (50.0%)	
Immunotherapy protocols, no. (%)			0.872
Monotherapy	14 (24.6%)	15 (25.9%)	
Combination therapy	43 (75.4%)	43 (74.1%)	
Number of treatment lines, no. (%)			0.512
First line	26 (45.6%)	30 (51.7%)	
Second line or more	31 (54.4%)	28 (48.3%)	

no., number; ECOG, Eastern Cooperative Oncology Group.

a The Fisher’s exact test was used.

### High expression of PCSK9 in NSCLC tissues correlated with poor efficacy in advanced NSCLC patients treated with anti-PD-1 immunotherapy

**Figure 2:**
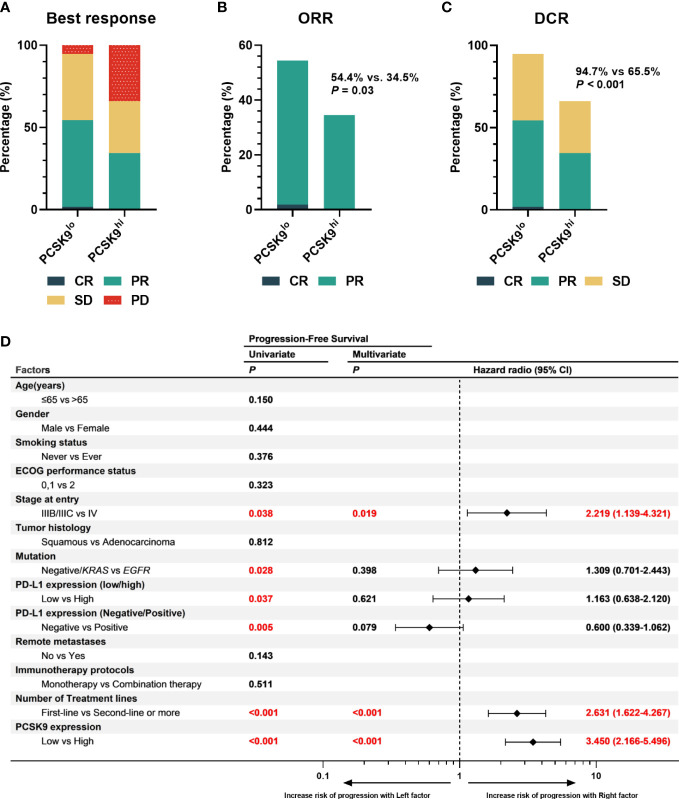
High expression of PCSK9 in NSCLC tissues was associated with poorer efficacy of anti-PD-1 immunotherapy in advanced NSCLC. **(A)** Best response of the PCSK9^hi^ and PCSK9^lo^ groups. **(B)** ORR of the PCSK9^hi^ and PCSK9^lo^ groups. **(C)** DCR of the PCSK9^hi^ and PCSK9^lo^ groups. **(D)** Univariate and multivariate analyses of factors associated with PFS. PD-L1 negative, TPS < 1%; PD-L1 low, TPS < 50%; PD-L1 high, TPS ≥ 50%.

One of the 57 patients in the low PCSK9 group achieved a complete response (CR), while no patients in the high PCSK9 group reached CR. In contrast with the higher progressive disease (PD) rate (20/58, 34.5%) of the high PCSK9 group, 31 of the 57 patients in the low PCSK9 group achieved a response (CR, *n* = 1; PR, *n* = 30) and 23 additional patients had stable disease (SD) with an ORR of 54.4% and a DCR of 94.7%, which were statistically significant with the ORR (34.5%, *P* = 0.03) and DCR (65.5%, *P <* 0.001) of the high PCSK9 group (**Table 2**; **Figures 2A–C**). Univariate analysis showed that PFS was associated with stage (*P* = 0.038), mutation (*P* = 0.028), low/high PD-L1 expression (*P* = 0.037), negative/positive PD-L1 expression (*P* = 0.005), the number of treatment lines (*P <* 0.001), and PCSK9 expression (*P <* 0.001). Multivariate analysis was used to adjust for the confounders of these factors, which indicated that PFS was associated with stage (HR, 2.219; 95% CI, 1.139–4.321; *P* = 0.019), the number of treatment lines (HR, 2.631; 95% CI, 1.622–4.267; *P <* 0.001), and PCSK9 expression (HR, 3.450; 95% CI, 2.166–5.496; *P <* 0.001) (**Figure 2D**). Subgroup analysis of PFS also showed that there was an increased risk of progression with PCSK9^hi^ in almost all the subgroups except ECOG PS = 2, stage IIIB/IIIC, and first-line treatment (**Supplementary Figure 2**). OS was associated with ECOG performance status (*P* = 0.011), immunotherapy protocols (*P =* 0.029), and PCSK9 expression (*P =* 0.009) in the univariate analysis, while the multivariate analysis showed that OS was only associated with PCSK9 expression in all these factors (21.4 *vs*. NR months; HR, 2.164; 95% CI, 1.190–3.935; *P =* 0.011) (**Supplementary Figure 3**). Nonetheless, a limitation should be considered for this OS result where 60% of the patients (69) did not reach the endpoint (42.3% of the patients were still alive and 17.4% were lost to follow-up), and a longer follow-up time for OS was required to confirm this result. In summary, high PCSK9 expression in NSCLC tissue was related to a worse response to these treatments, which was also an independent risk factor for both PFS and OS in advanced NSCLC patients with anti-PD-1 immunotherapy.

**Table 2:** Best response of advanced NSCLC patients in the PCSK9^lo^ and PCSK9^hi^ groups to anti-PD-1 immunotherapy.

	PCSK9^lo^ (*n* = 57)	PCSK9^hi^ (*n* = 58)	*P* ^a^
Best response, no. (%)			
CR	1 (1.8%)	0 (0%)	
PR	30 (52.6%)	20 (34.5%)	
SD	23 (40.4%)	18 (31.0%)	
PD	3 (5.3%)	20 (34.5%)	
ORR, no. (%)	31 (54.4%)	20 (34.5%)	0.03
DCR, no. (%)	54 (94.7%)	38 (65.5%)	<0.001

no., number; CR, complete response; PR, partial response; SD, stable disease; PD, progressive disease; ORR, objective response rate; DCR, disease control rate.

a The chi-square test was used.

### High expression of PCSK9 correlated with poor lymphocyte infiltration

**Figure 3:**
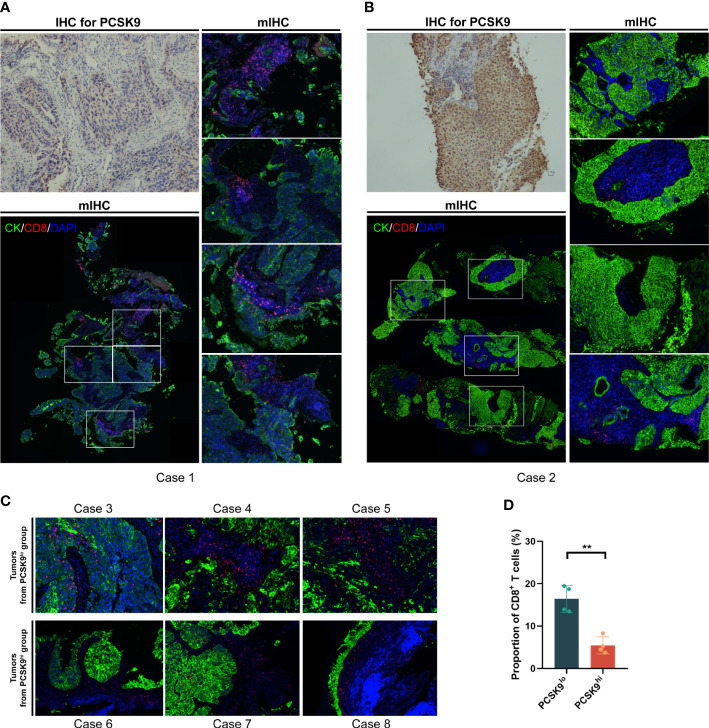
CD8^+^ T cells increased and moved into the tumor-cell-rich areas in PCSK9^lo^ tissue, while they stayed in the margin of tumor areas in PCSK9^hi^ tissue. **(A, B)** Multiplexed immunofluorescent staining of CK, CD8, and DAPI in the tumor microenvironment (bottom left panel and right panel, ×200 original magnification) of PCSK9^lo^ **(A)** or PCSK9^hi^ **(B)** tissue adjudicated by immunohistochemistry (upper left panel, ×200 magnification). **(C)** Typical views of other tumors from the PCSK9^lo^ or PCSK9^hi^ groups (×200 magnification). **(D)** Quantitative estimates of the density of CD8^+^ T cells: proportion of CD8 = CD8 number/DAPI number. **P < 0.01.

As previously reported by Liu et al. (7), PCSK9 caused a decrease in surface MHC I levels, which had a strong influence on MHC I presentation of peptide antigens, thus leading to attenuation of intratumoral T-cell infiltration (notably CD8^+^ T cells) *in vivo* and *in vitro*. To further confirm whether this relationship existed in NSCLC tissue between PCSK9 expression with density and distribution of intratumoral CD8^+^ T cells, we examined tumor cells and CD8^+^ T cells in PCSK9^hi^ and PCSK9^lo^ NSCLC tissues by multiplexed immunofluorescent staining (**Figure 3**). There was a greater increase of CD8^+^ T cells in PCSK9^lo^ tissue than in PCSK9^hi^ tissue. Furthermore, a trend was observed that a large proportion of CD8^+^ T cells moved into tumor-cell-rich areas in PCSK9^lo^ tissue, while they stayed in the margin of tumor areas in PCSK9^hi^ tissue.

### Tumor growth was attenuated with PCSK9 inhibition in combination with CD137 costimulation in syngeneic mice

**Figure 4:**
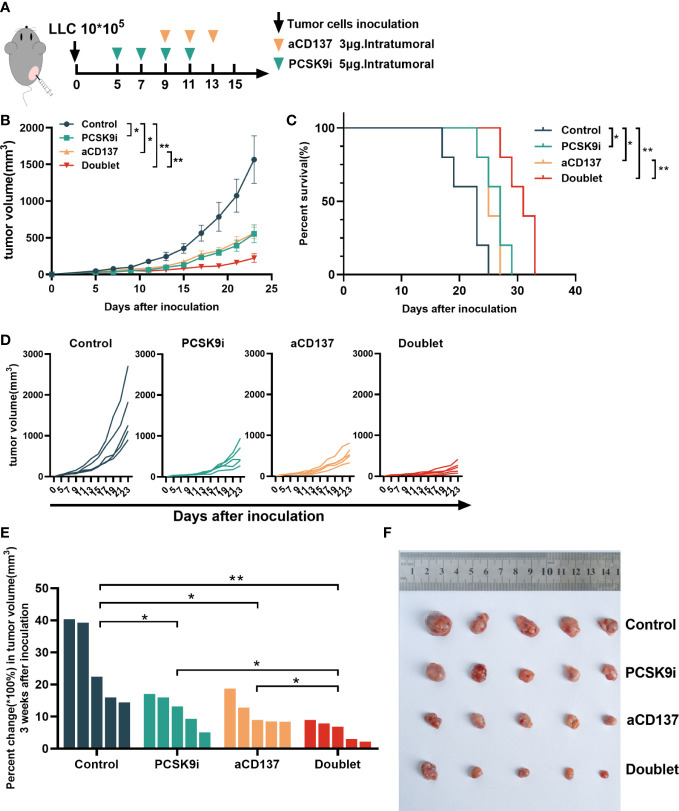
Tumor growth was attenuated with PCSK9 inhibition in combination with CD137 costimulation in syngeneic mice. **(A)** Experimental protocol. *n* = 5 mice per group. Intratumoral. **(B)** Tumor growth curve of each group (*P*-value was calculated by two-way ANOVA). **(C)** Overall survival (log-rank test). **(D)** Tumor growth curve of each mouse. **(E)** Percent change in tumor volume on day 21 compared with day 5 (baseline) after inoculation (unpaired *t*-test). **(F)** Tumors harvested on day 21. Data shown as mean ± SEM. *P < 0.05, **P < 0.01. LLC, Lewis lung carcinoma cells; PCSK9i, PCSK9 inhibitor; aCD137, anti-CD137 agonist; Doublet, the group treated with the PCSK9 inhibitor and the anti-CD137 agonist.

To evaluate the synergistic effect of PCSK9 inhibition and CD137 costimulation in tumor growth, syngeneic mouse models were created by generating subcutaneous LLC cells for tumor growth delay experiments with the PCSK9 neutralizing antibody evolocumab and the anti-mouse CD137 agonist antibody 1D8 (**Figure 4A**). The PCSK9 inhibitor and the anti-CD137 agonist alone delayed the growth of tumors. Moreover, tumor growth was further delayed by the PCSK9 inhibitor in combination with the CD137 agonist with long-term survival of the host mice in the doublet group (**Figures 4B–D**). The combination of the PCSK9 inhibitor with the anti-CD137 agonist led to greater tumor volume control at 21 days after inoculation from day 5 (baseline prior to antibody injection) compared with the two single groups (**Figures 4E, F**).

**Figure 5:**
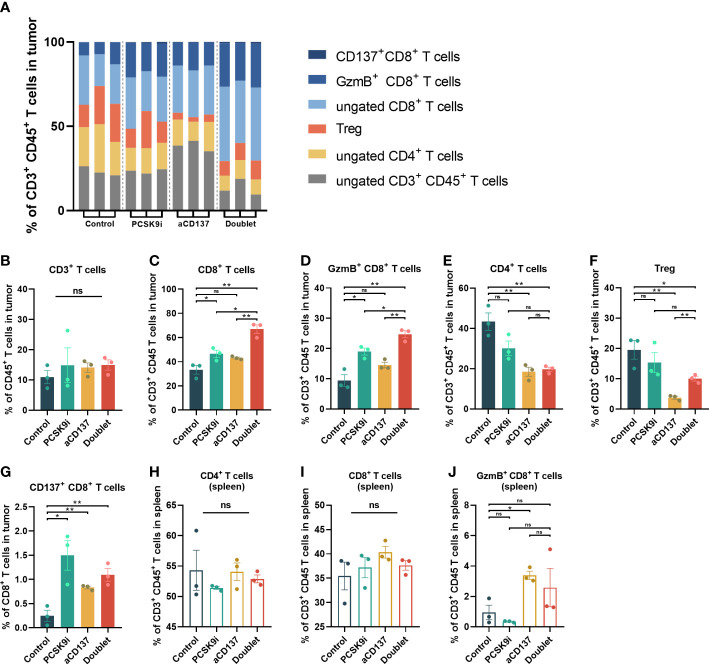
The proportion of CD8^+^ and GzmB^+^ CD8^+^ T cells increased significantly in the tumors of the doublet group with a reduction of Tregs. **(A)** Frequency of tumor-infiltrating CD4^+^ and CD8^+^ T cells of CD3^+^CD45^+^ leukocytes in LLC tumors of each group on day 21 (*n* = 3 per group). **(B–G)** Quantitative estimate of various immune effector cells in 1 * 10^6^ immune cells of tissue in each group determined by flow cytometry. *n* = 3 tumors per group (*P*-value was calculated by unpaired *t*-test or the Mann–Whitney test). **(H–J)** Quantitative estimate of CD4^+^, CD8^+^, and GzmB^+^ CD8^+^ T cells in 0.1 * 10^6^ immune cells of the spleen in each group determined by flow cytometry. *n* = 3 tumors per group (*P*-value was calculated by unpaired *t*-test or the Mann–Whitney test). *ns, No significant difference, *P < 0.05, **P < 0.01.

To determine whether this combination was synergistic with the change in immune cell infiltration, we performed additional analyses of TIL variation by flow cytometry on day 21 after inoculation. No noticeable change in CD3^+^CD45^+^ T cells was observed, but the proportion of CD4^+^ and CD8^+^ T cells varied widely (**Figures 5A, B**). The percentage of CD8^+^ T cells increased significantly in the PCSK9i group than in the control group, and it was the highest in the doublet group (**Figure 5C**). More importantly, similar trends were observed for granzyme B^+^ (GzmB^+^) CD8^+^ T cells (**Figure 5D**). The percentage of CD4^+^ T cells and Tregs decreased significantly in the aCD137 group and the doublet group than in the control group (**Figures 5E, F**). The fraction of CD137^+^CD8^+^ T cells was significantly elevated in the PCSK9i group (**Figure 5G**). No similar trends of the percentage of CD4^+^, CD8^+^, and GzmB^+^ CD8^+^ T cells were observed in the spleens of the host mice (**Figures 5H–J**).

**Figure 6:**
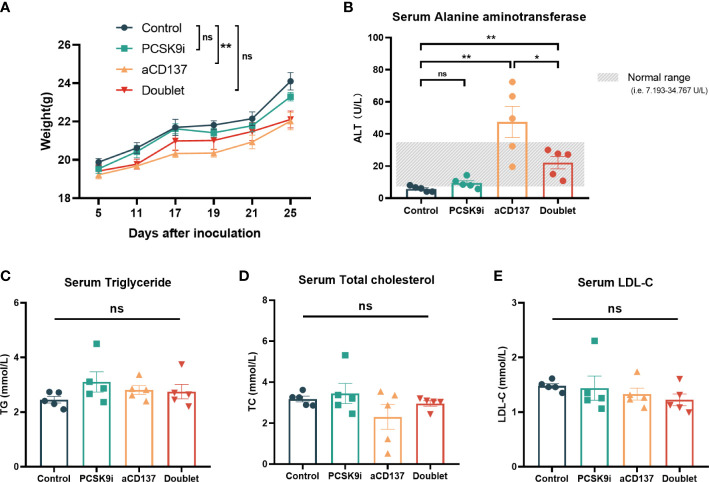
Body weight and serological changes of the mouse models after the treatments. **(A)** Body weight change of mice in each group (*n* = 5 per group, two-way ANOVA). **(B–E)** Serum ALT, TG, TCHO, and LDL-C levels of mice in each group on day 21 (*n* = 5 per group, unpaired *t*-test or the Mann–Whitney test). *ns, No significant difference, *P < 0.05, **P < 0.01.

Considering the toxicity of the CD137 agonist antibody, the body weights of mice were monitored throughout the treatment period. Although no weight loss was observed in each mouse during treatment, the weight of mice in the anti-CD137 group increased slowly compared with the other groups (**Figure 6A**). Additionally, the levels of serum ALT (also known as serum glutamic-pyruvic transaminase) were increased in the anti-CD137 group and the doublet group. However, remarkably, the levels of serum ALT were lower in the doublet group than in the aCD137 group, which were within the normal range (**Figure 6B**). Furthermore, there was no significance in serum triglyceride, total cholesterol, and LDL-C (**Figures 6C–E**).

## Discussion

In our work, we investigated PCSK9 expression by immunohistochemistry in tumor tissue from advanced NSCLC patients before receiving anti-PD-1 immunotherapy. Our work demonstrated that aside from being related to poor response, high PCSK9 expression in baseline NSCLC tissues was also an independent risk factor for both PFS and OS in advanced NSCLC patients with anti-PD-1 immunotherapy.

In a study by Liu et al. (7) published in *Nature*, PCSK9 promotes lysosome-mediated degradation of MHC I in tumor cells. The recycling process of MHC I back to the membrane was inhibited, thereby resulting in the evasion of cytotoxic T-cell (CTL) surveillance and finally leading to the reduction of lymphocyte infiltration and tumor immune evasion. CD8^+^ CTLs were identified as the most critical for the antitumor effect of PCSK9 deficiency in the trial with immunodeficient mice. The type, density, and location of immune cells within the tumor microenvironment (TME) were reported to affect the efficacy of anti-PD-1/PD-L1-based immunotherapy, which led to a concept called “hot” (highly infiltrated) and “cold” (poorly infiltrated) immune tumors, an unofficial classification of tumors that has been increasingly advocated (12). A tumor with a “hot” TME was associated with an increased response to anti-PD1/PD-L1-based immunotherapy (13, 14). Conversely, a “cold” tumor was characterized with features such as a low presence of T cells and an immunosuppressive tumor infiltrate which would support tumor growth and cause a limited response to a checkpoint blockade (15, 16). Additionally, low expression of antigen presentation machinery makers such as MHC I also contributed to the formation of “cold” tumors (17). Altogether, reduced CD8^+^ T-cell infiltration—caused by PCSK9-mediated deficiency of the tumor antigen peptide–MHC that could be targets for cytotoxic T-cell recognition—might be one of the reasons why patients with PCSK9^lo^ tumor tissue were more likely to benefit from ICIs compared with those with PCSK9^hi^ tumor under advanced NSCLC treatment.

To confirm whether this phenomenon existed in NSCLC tumor tissue between different PCSK9 expression levels and CD8^+^ T cells, we next performed multiplexed immunofluorescent staining to show tumor cells and CD8^+^ T cells in NSCLC tissue with different PCSK9 expression levels. A trend was observed that CD8^+^ T cells were reduced and almost located in the margin of tumor areas in the PCSK9^hi^ tissue, while they were more concentrated in the tumor-center areas with an increased number in PCSK9^lo^ tissue, which was in agreement with the conclusion of a previous research by Liu et al. (7) using PCSK9 depletion.

Given the negative regulation of PCSK9 on T-cell recruitment and activation, the value of PCSK9 as a potential target for antitumor combination therapeutics was worth further exploring. Liu et al. (7) showed that the response to anti-PD-1 treatment *in vivo* was enhanced by PCSK9 deletion and inhibition by evolocumab and alirocumab, two PCSK9-neutralizing antibodies. Considering the trigger function of MHC I antigen presentation in T-cell activation, there might be other therapeutic combination strategies with PCSK9 inhibition based on the process of T-cell activation. Three signals were required in the process of T-cell activation: antigen peptide MHC–T-cell receptor (TCR) signaling, costimulatory signaling, and cytokine support (18, 19). PCSK9 disturbed the antigen peptide MHC–TCR signaling, the “first signal” required in the initiation of specific T-cell activation. Costimulatory signaling, the “second signal,” was required in the following process of T-cell activation, which could regulate T-cell differentiation, effector function, survival, and memory formation (20–22). The function of costimulatory signals also largely depended on antigen recognition and TCR signaling (23). Next, we explored the potential synergistic antitumor effect by PCSK9 inhibition with CD137 costimulation.

Syngeneic mouse models were built in our research. In consensus with previous reports (7, 24), tumor growth delay was observed with the PCSK9 inhibitor evolocumab and the anti-CD137 agonist 1D8 alone. Moreover, in our research, tumor growth was further delayed by PCSK9 inhibition in combination with the CD137 agonist with long-term survival of the host mice in the doublet group. Further analysis of TILs showed that 1) the proportion of CD8^+^ and GzmB^+^ CD8^+^ T cells was remarkably elevated in the doublet group. These results suggested the synergistic effect on T-cell activation from the two signals caused by PCSK9 inhibition and CD137 costimulation. PCSK9 inhibition represented a mechanism to preserve antigen presentation by positive regulation of MHC I, which might provide robust TCR signals for the following costimulatory signals triggered by the anti-CD137 agonist. CD137 expression was highly antigen-induced, especially in the subset of CD8^+^ T cells, which was reported in previous studies (25, 26). The combined application with the CD137 agonist effectively increased the number of tumor-related CD8^+^ T cells and exerted its antitumor effect. 2) Elimination of Tregs was observed in the doublet group, similar to the aCD137 group, which was also reported by Freeman et al. (27) and our previous study (11). The reduction of Tregs was probably because of the antibody-mediated cell-dependent phagocytosis (ADCP) of Tregs, mediated by the activation of macrophages by IgG-Fc (28–30). The loss of Tregs caused by CD137 mAb could reverse the immunosuppressive environment and simultaneously affect CD8^+^ T cells in the tumor while also allowing new CD8^+^ T cells to infiltrate the TME (27). Altogether, these results demonstrated that PCSK9 inhibition enhanced immunogenicity and T-cell recruitment. The use of the CD137 agonist could further promote CD8^+^ T-cell expansion and activation and also relieve the immunosuppressive effects from Tregs, resulting in a synergistic antitumor effect. Similar combination strategies with the CD137 agonist to enhance efficacy have received considerable attention since the early discovery of the CD137 agonist (31–33), such as chemotherapy (34, 35), radiotherapy (36, 37), or cancer vaccines (38, 39), possibly through the enhanced immunogenicity with more tumor antigen cross-presentation to improve the efficacy of the immune checkpoint inhibitors (40).

Urelumab, a CD137 agonist antibody, showed treatment-related severe adverse effects in the form of liver inflammation during phase I and II trials (41). Hepatotoxicity has been a concern in all studies conducted on the CD137 agonist antibody. At present, several strategies have been used in previous studies to reduce toxicity: a CD137-based antibody engaged with other specific molecule which specifically brings CD137 agonism to the tumor tissue microenvironment (42), an antibody with Fc lacking trimeric CD137 constructs to avoid FcɣR binding-mediated liver toxicity (43), intratumoral injections of low doses of CD137 mAb (44), and dose reduction with a combination of other immune-modulating agents (45). In our work, although a low-dose intratumoral injection was performed, weight growth stunting and an increase in ALT were still observed in the anti-CD137 group. Although the levels of ALT were still higher in the doublet group than those in the control group, the levels reduced to a normal range. It was reported that both serum ALT and AST were attenuated by PCSK9 inhibition therapy in alcoholic liver disease and non-alcoholic fatty liver disease (NAFLD) (46, 47), but no previous studies reported the attenuation of aminotransferase by PCSK9 inhibitors in tumor treatment-related hepatotoxicity. PCSK9 inhibitors have shown a neutral effect on liver function (48), which was one of the reasons why we chose the combination of the PCSK9 inhibitor and the anti-CD137 agonist in our work aside from the possible synergy on T-cell activation mentioned above. The therapeutic strategy of PCSK9 inhibition with CD137 costimulation would have potential implications for future research and clinical practice.

The change in serum lipids was not observed in the treatment groups in our work probably because of intratumoral injections and low doses of evolocumab which were not enough to affect the lipid metabolism of the host mice.

Our research has limitations. First, although our research obtained some preclinical findings, prospective studies are still needed to confirm the value of PCSK9 as a predictive marker. Second, some outcomes might show bias in subgroups with small numbers of patients, such as in the subgroup with ECOG PS = 2 and in EGFR-positive patients. A larger population is needed to further verify this conclusion. Third, although the current results have shown a trend that high PCSK9 expression was an independent risk factor for OS, it still requires a further follow-up and an update on OS data because 69 patients (49 were still alive and 20 were lost to follow-up) did not meet the events for OS at the end of the follow-up period. Fourth, based on the mechanism of PCSK9 regulation on MHC I and the importance of protein level information in clinical decision-making, our research was committed to exploring the value of PCSK9 as an immunohistochemical marker at the protein level by IHC. However, some methods, such as RNA sequencing, could be more sensitive. Verification of multiple tests is necessary in future studies. Finally, within the context of local regulation of PCSK9 and reduced toxicity of CD137 local agonism mentioned above, intratumoral injection was used in our animal experiment to explore the synergistic mechanism in the local tumor area. This might be a limitation for clinical practice from a translational perspective because systemic medication was a major strategy in advanced NSCLC treatments. The methods for drug use and trials for dose and dosage form should be explored in future research from a practical standpoint.

To conclude, our study demonstrated that high PCSK9 expression in baseline tumor tissue could be a deleterious factor in advanced NSCLC patients receiving ICIs with poorer efficacy. Additionally, the PCSK9 inhibitor combined with CD137 costimulation could slow tumor growth with enhanced recruitment of CD8^+^ and GzmB^+^ CD8^+^ T cells and reduction of Tregs in a syngeneic mouse model, providing a novel perspective of therapeutic strategies for future research and clinical practice.

## Data availability statement

The raw data supporting the conclusions of this article will be made available by the authors, without undue reservation.

## Ethics statement

The studies involving human participants were reviewed and approved by the Clinical Research Ethics Committee of Beijing Chest Hospital, Capital Medical University, Beijing, China. The Ethics Committee waived the requirement of written informed consent for participation. The animal study was reviewed and approved by the Animal Use and Care Committee of Beijing Chest Hospital.

## Author contributions

XG, JW, SZ, and HZ conceived the work, developed the methodology, and interpreted the data. XG, LY, CJ, SL, XW, and BY performed/arranged the experiments. XG analyzed the data and wrote the manuscript. WL was the consultant for the animal experiments. NC was the consultant for immunohistochemistry and provided the pathology laboratory. JW, SZ, and HZ provided financial support for the project. All authors contributed to the article and approved the submitted version.

## Supplementary material

Supplementary Figure 1 Relationship between PCSK9 expression in NSCLC tissues and clinicopathological characteristics of advanced NSCLC patients. ECOG, Eastern Cooperative Oncology Group. *PD-L1 negative, TPS<1%; PD-L1 low, TPS<50%; PD-L1 high, TPS≥50%.

Supplementary Figure 2 Subgroup analysis for PFS of PCSK9^hi^ and PCSK9^lo^ group. *PD-L1 negative, TPS<1%; PD-L1 low, TPS<50%; PD-L1 high, TPS≥50%.

Supplementary Figure 3 PCSK9 was an independent risk factor for OS in advanced NSCLC patients with anti-PD1 immunotherapy. **(A).** Kaplan-Meier graph for overall survival in the population of PCSK9^hi^ and PCSK9^lo^ group. **(B).** Univariate and multivariate analyses of factors associated with overall survival. Data cutoff was April 30, 2022. *PD-L1 negative, TPS<1%; PD-L1 low, TPS<50%; PD-L1 high, TPS≥50%.

## References

[1] Abifadel M Guerin M Benjannet S Rabes JP Le Goff W Julia Z. Identification and characterization of new gain-of-function mutations in the PCSK9 gene responsible for autosomal dominant hypercholesterolemia. Atherosclerosis (2012) 223:394–400. doi: 10.1016/j.atherosclerosis.2012.04.006 22683120

[2] Xu B Li S Fang Y Zou Y Song D Zhang S. Proprotein convertase Subtilisin/Kexin type 9 promotes gastric cancer metastasis and suppresses apoptosis by facilitating MAPK signaling pathway through HSP70 up-regulation. Front Oncol (2020) 10:609663. doi: 10.3389/fonc.2020.609663 33489919 PMC7817950

[3] Zhang SZ Zhu XD Feng LH Li XL Liu XF Sun HC. PCSK9 promotes tumor growth by inhibiting tumor cell apoptosis in hepatocellular carcinoma. Exp Hematol Oncol (2021) 10:25. doi: 10.1186/s40164-021-00218-1 33789749 PMC8011384

[4] Sun X Essalmani R Day R Khatib AM Seidah NG Prat A. Proprotein convertase subtilisin/kexin type 9 deficiency reduces melanoma metastasis in liver. Neoplasia (2012) 14:1122–31. doi: 10.1593/neo.121252 PMC3540939 23308045

[5] Piao MX Bai JW Zhang PF Zhang YZ. PCSK9 regulates apoptosis in human neuroglioma u251 cells *via* mitochondrial signaling pathways. Int J Clin Exp Pathol (2015) 8:2787–94. PMC4440094 26045785

[6] Xu X Cui Y Cao L Zhang Y Yin Y Hu X. PCSK9 regulates apoptosis in human lung adenocarcinoma A549 cells *via* endoplasmic reticulum stress and mitochondrial signaling pathways. Exp Ther Med (2017) 13:1993–9. doi: 10.3892/etm.2017.4218 PMC5443240 28565798

[7] Liu X Bao X Hu M Chang H Jiao M Cheng J. Inhibition of PCSK9 potentiates immune checkpoint therapy for cancer. Nature (2020) 588:693–8. doi: 10.1038/s41586-020-2911-7 PMC7770056 33177715

[8] Bonaventura A Grossi F Carbone F Vecchie A Minetti S Bardi N. Serum PCSK9 levels at the second nivolumab cycle predict overall survival in elderly patients with NSCLC: a pilot study. Cancer Immunol Immunother (2019) 68:1351–8. doi: 10.1007/s00262-019-02367-z PMC11028217 31327024

[9] Xie M Yu X Chu X Xie H Zhou J Zhao J. Low baseline plasma PCSK9 level is associated with good clinical outcomes of immune checkpoint inhibitors in advanced non-small cell lung cancer. Thorac Cancer (2022) 13:353–60. doi: 10.1111/1759-7714.14259 PMC8807327 34962050

[10] Camp RL Dolled-Filhart M Rimm DL. X-Tile: a new bio-informatics tool for biomarker assessment and outcome-based cut-point optimization. Clin Cancer Res (2004) 10:7252–9. doi: 10.1158/1078-0432.CCR-04-0713 15534099

[11] Yi L Jin X Wang J Yan Z Cheng X Wen T. CD137 agonists targeting CD137-mediated negative regulation show enhanced antitumor efficacy in lung cancer. Front Immunol (2022) 13:771809. doi: 10.3389/fimmu.2022.771809 35197968 PMC8859117

[12] Galon J Bruni D. Approaches to treat immune hot, altered and cold tumours with combination immunotherapies. Nat Rev Drug Discovery (2019) 18:197–218. doi: 10.1038/s41573-018-0007-y 30610226

[13] Tumeh PC Harview CL Yearley JH Shintaku IP Taylor EJ Robert L. PD-1 blockade induces responses by inhibiting adaptive immune resistance. Nature (2014) 515:568–71. doi: 10.1038/nature13954 PMC4246418 25428505

[14] Taube JM. Unleashing the immune system: PD-1 and PD-ls in the pre-treatment tumor microenvironment and correlation with response to PD-1/PD-L1 blockade. Oncoimmunology (2014) 3:e963413. doi: 10.4161/21624011.2014.963413 25914862 PMC4292419

[15] Wachsmann MB Pop LM Vitetta ES. Pancreatic ductal adenocarcinoma: A review of immunologic aspects. J Investig Med (2012) 60:643–63. doi: 10.2310/JIM.0b013e31824a4d79 PMC3319488 22406516

[16] Belmontes B Sawant DV Zhong W Tan H Kaul A Aeffner F. Immunotherapy combinations overcome resistance to bispecific T cell engager treatment in T cell-cold solid tumors. Sci Transl Med (2021) 13. doi: 10.1126/scitranslmed.abd1524 34433637

[17] Hegde PS Karanikas V Evers S. The where, the when, and the how of immune monitoring for cancer immunotherapies in the era of checkpoint inhibition. Clin Cancer Res (2016) 22:1865–74. doi: 10.1158/1078-0432.CCR-15-1507 27084740

[18] Smith-Garvin JE Koretzky GA Jordan MS. T Cell activation. Annu Rev Immunol (2009) 27:591–619. doi: 10.1146/annurev.immunol.021908.132706 19132916 PMC2740335

[19] Neefjes J Jongsma ML Paul P Bakke O. Towards a systems understanding of MHC class I and MHC class II antigen presentation. Nat Rev Immunol (2011) 11:823–36. doi: 10.1038/nri3084 22076556

[20] Esensten JH Helou YA Chopra G Weiss A Bluestone JA. CD28 costimulation: From mechanism to therapy. Immunity (2016) 44:973–88. doi: 10.1016/j.immuni.2016.04.020 PMC4932896 27192564

[21] Pollok KE Kim YJ Zhou Z Hurtado J Kim KK Pickard RT. Inducible T cell antigen 4-1BB. analysis of expression and function. J Immunol (1993) 150:771–81. doi: 10.4049/jimmunol.150.3.771 7678621

[22] Zhou AC Wagar LE Wortzman ME Watts TH. Intrinsic 4-1BB signals are indispensable for the establishment of an influenza-specific tissue-resident memory CD8 T-cell population in the lung. Mucosal Immunol (2017) 10:1294–309. doi: 10.1038/mi.2016.124 28051085

[23] Croft M. The role of TNF superfamily members in T-cell function and diseases. Nat Rev Immunol (2009) 9:271–85. doi: 10.1038/nri2526 PMC2737409 19319144

[24] Etxeberria I Bolanos E Teijeira A Garasa S Yanguas A Azpilikueta A. Antitumor efficacy and reduced toxicity using an anti-CD137 probody therapeutic. Proc Natl Acad Sci U.S.A. (2021) 118(26):e2025930118. doi: 10.1073/pnas.2025930118 34172583 PMC8255787

[25] Weigelin B Bolanos E Teijeira A Martinez-Forero I Labiano S Azpilikueta A. Focusing and sustaining the antitumor CTL effector killer response by agonist anti-CD137 mAb. Proc Natl Acad Sci U.S.A. (2015) 112:7551–6. doi: 10.1073/pnas.1506357112 PMC4475992 26034288

[26] Ye Z Hellstrom I Hayden-Ledbetter M Dahlin A Ledbetter JA Hellstrom KE. Gene therapy for cancer using single-chain fv fragments specific for 4-1BB. Nat Med (2002) 8:343–8. doi: 10.1038/nm0402-343 11927939

[27] Freeman ZT Nirschl TR Hovelson DH Johnston RJ Engelhardt JJ Selby MJ. A conserved intratumoral regulatory T cell signature identifies 4-1BB as a pan-cancer target. J Clin Invest (2020) 130:1405–16. doi: 10.1172/JCI128672 PMC7269585 32015231

[28] Ha D Tanaka A Kibayashi T Tanemura A Sugiyama D Wing JB. Differential control of human treg and effector T cells in tumor immunity by fc-engineered anti-CTLA-4 antibody. Proc Natl Acad Sci U.S.A. (2019) 116:609–18. doi: 10.1073/pnas.1812186116 PMC6329979 30587582

[29] Dahan R Sega E Engelhardt J Selby M Korman AJ Ravetch JV. FcgammaRs modulate the anti-tumor activity of antibodies targeting the PD-1/PD-L1 axis. Cancer Cell (2015) 28:285–95. doi: 10.1016/j.ccell.2015.08.004 26373277

[30] Marson A Kretschmer K Frampton GM Jacobsen ES Polansky JK Macisaac KD. Foxp3 occupancy and regulation of key target genes during T-cell stimulation. Nature (2007) 445:931–5. doi: 10.1038/nature05478 PMC3008159 17237765

[31] Melero I Sanmamed MF Glez-Vaz J Luri-Rey C Wang J Chen L. CD137 (4-1BB)-Based cancer immunotherapy on its 25th anniversary. Cancer Discovery (2023) 13:1–19. doi: 10.1158/2159-8290.CD-22-1029 36576322

[32] Perez-Gracia JL Labiano S Rodriguez-Ruiz ME Sanmamed MF Melero I. Orchestrating immune check-point blockade for cancer immunotherapy in combinations. Curr Opin Immunol (2014) 27:89–97. doi: 10.1016/j.coi.2014.01.002 24485523

[33] Melero I Berman DM Aznar MA Korman AJ Perez Gracia JL Haanen J. Evolving synergistic combinations of targeted immunotherapies to combat cancer. Nat Rev Cancer (2015) 15:457–72. doi: 10.1038/nrc3973 26205340

[34] Kim YH Choi BK Oh HS Kang WJ Mittler RS Kwon BS. Mechanisms involved in synergistic anticancer effects of anti-4-1BB and cyclophosphamide therapy. Mol Cancer Ther (2009) 8:469–78. doi: 10.1158/1535-7163.MCT-08-0993 19190115

[35] Kim YH Choi BK Kim KH Kang SW Kwon BS. Combination therapy with cisplatin and anti-4-1BB: synergistic anticancer effects and amelioration of cisplatin-induced nephrotoxicity. Cancer Res (2008) 68:7264–9. doi: 10.1158/0008-5472.CAN-08-1365 PMC2551756 18794112

[36] Verbrugge I Hagekyriakou J Sharp LL Galli M West A Mclaughlin NM. Radiotherapy increases the permissiveness of established mammary tumors to rejection by immunomodulatory antibodies. Cancer Res (2012) 72:3163–74. doi: 10.1158/0008-5472.CAN-12-0210 22570253

[37] Rodriguez-Ruiz ME Rodriguez I Garasa S Barbes B Solorzano JL Perez-Gracia JL. Abscopal effects of radiotherapy are enhanced by combined immunostimulatory mAbs and are dependent on CD8 T cells and crosspriming. Cancer Res (2016) 76:5994–6005. doi: 10.1158/0008-5472.CAN-16-0549 27550452

[38] Ito F Li Q Shreiner AB Okuyama R Jure-Kunkel MN Teitz-Tennenbaum S. Anti-CD137 monoclonal antibody administration augments the antitumor efficacy of dendritic cell-based vaccines. Cancer Res (2004) 64:8411–9. doi: 10.1158/0008-5472.CAN-04-0590 15548712

[39] Bartkowiak T Singh S Yang G Galvan G Haria D Ai M. Unique potential of 4-1BB agonist antibody to promote durable regression of HPV+ tumors when combined with an E6/E7 peptide vaccine. Proc Natl Acad Sci U.S.A. (2015) 112:E5290–5299. doi: 10.1073/pnas.1514418112 PMC4586868 26351680

[40] Salas-Benito D Perez-Gracia JL Ponz-Sarvise M Rodriguez-Ruiz ME Martinez-Forero I Castanon E. Paradigms on immunotherapy combinations with chemotherapy. Cancer Discovery (2021) 11:1353–67. doi: 10.1158/2159-8290.CD-20-1312 33712487

[41] Sznol M Hodi FS Margolin K Mcdermott DF Ernstoff MS Kirkwood JM. Phase I study of BMS-663513, a fully human anti-CD137 agonist monoclonal antibody, in patients (pts) with advanced cancer (CA). J Clin Oncol (2008) 26:3007–7. doi: 10.1200/jco.2008.26.15_suppl.3007

[42] Hinner MJ Aiba RSB Jaquin TJ Berger S Durr MC Schlosser C. Tumor-localized costimulatory T-cell engagement by the 4-1BB/HER2 bispecific antibody-anticalin fusion PRS-343. Clin Cancer Res (2019) 25:5878–89. doi: 10.1158/1078-0432.CCR-18-3654 31138587

[43] Compte M Harwood SL Munoz IG Navarro R Zonca M Perez-Chacon G. A tumor-targeted trimeric 4-1BB-agonistic antibody induces potent anti-tumor immunity without systemic toxicity. Nat Commun (2018) 9:4809. doi: 10.1038/s41467-018-07195-w 30442944 PMC6237851

[44] Palazon A Martinez-Forero I Teijeira A Morales-Kastresana A Alfaro C Sanmamed MF. The HIF-1alpha hypoxia response in tumor-infiltrating T lymphocytes induces functional CD137 (4-1BB) for immunotherapy. Cancer Discovery (2012) 2:608–23. doi: 10.1158/2159-8290.CD-11-0314 22719018

[45] Etxeberria I Glez-Vaz J Teijeira A Melero I. New emerging targets in cancer immunotherapy: CD137/4-1BB costimulatory axis. ESMO Open (2020) 4:e000733. doi: 10.1136/esmoopen-2020-000733 32611557 PMC7333812

[46] Lee JS Mukhopadhyay P Matyas C Trojnar E Paloczi J Yang YR. PCSK9 inhibition as a novel therapeutic target for alcoholic liver disease. Sci Rep (2019) 9:17167. doi: 10.1038/s41598-019-53603-6 31748600 PMC6868240

[47] Shafiq M Walmann T Nutalapati V Gibson C Zafar Y. Effects of proprotein convertase subtilisin/kexin type-9 inhibitors on fatty liver. World J Hepatol (2020) 12:1258–66. doi: 10.4254/wjh.v12.i12.1258 PMC7772734 33442452

[48] Rimbert A Smati S Dijk W Le May C Cariou B. Genetic inhibition of PCSK9 and liver function. JAMA Cardiol (2021) 6:353–4. doi: 10.1001/jamacardio.2020.5341 PMC7643040 33146683

