# Supplementary figures and images for: PCSK9 regulates the efficacy of immune checkpoint therapy in lung cancer
Source: Front Immunol. 2023 Mar 21;14:1142428. doi: 10.3389/fimmu.2023.1142428 (PMC10070680; doi:10.3389/fimmu.2023.1142428)

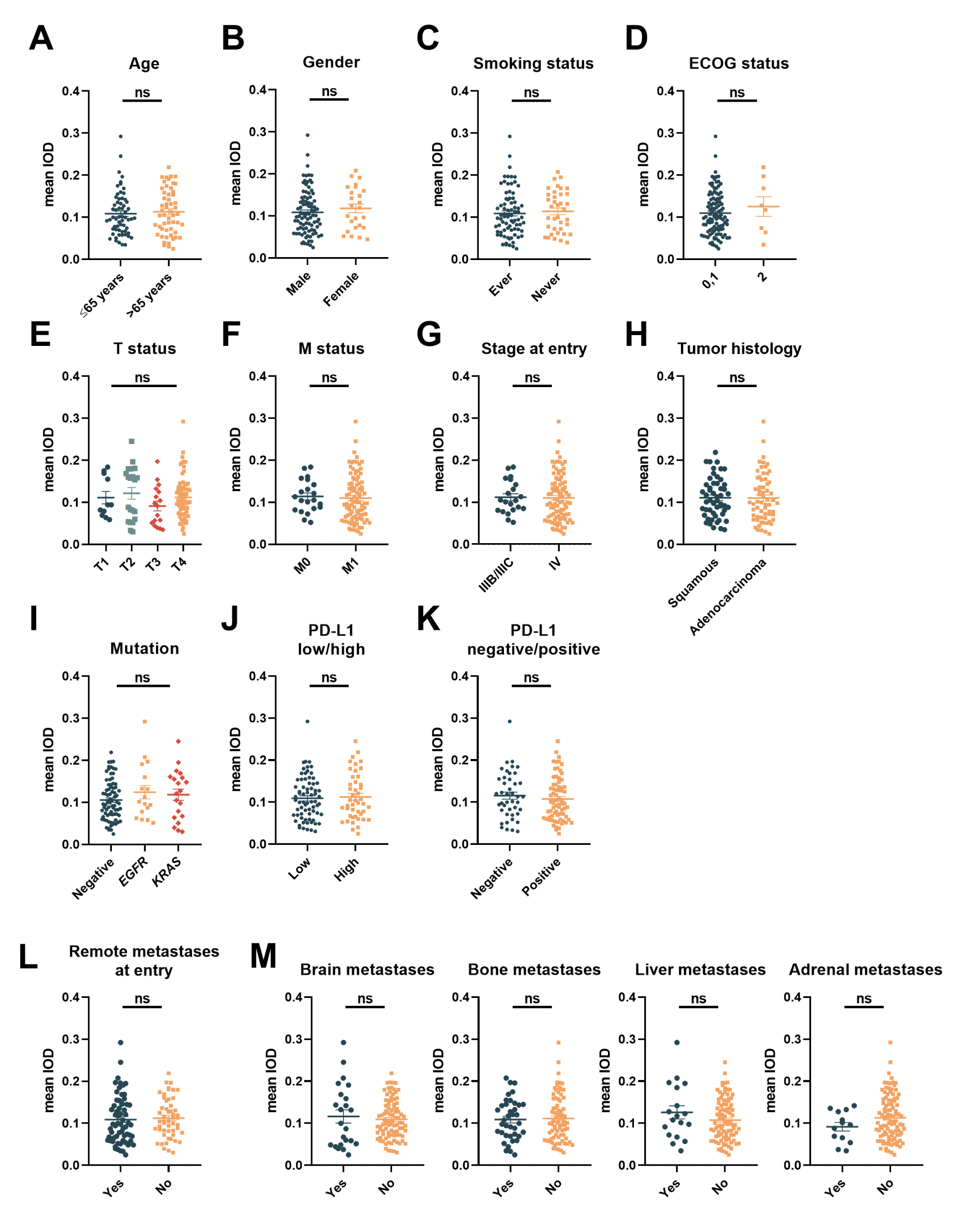

Supplement: Supplementary Figure 1 — Relationship between PCSK9 expression in NSCLC tissues and clinicopathological characteristics of advanced NSCLC patients. ECOG, Eastern Cooperative Oncology Group. *PD-L1 negative, TPS<1%; PD-L1 low, TPS<50%; PD-L1 high, TPS≥50%. [file Image_1.tif]

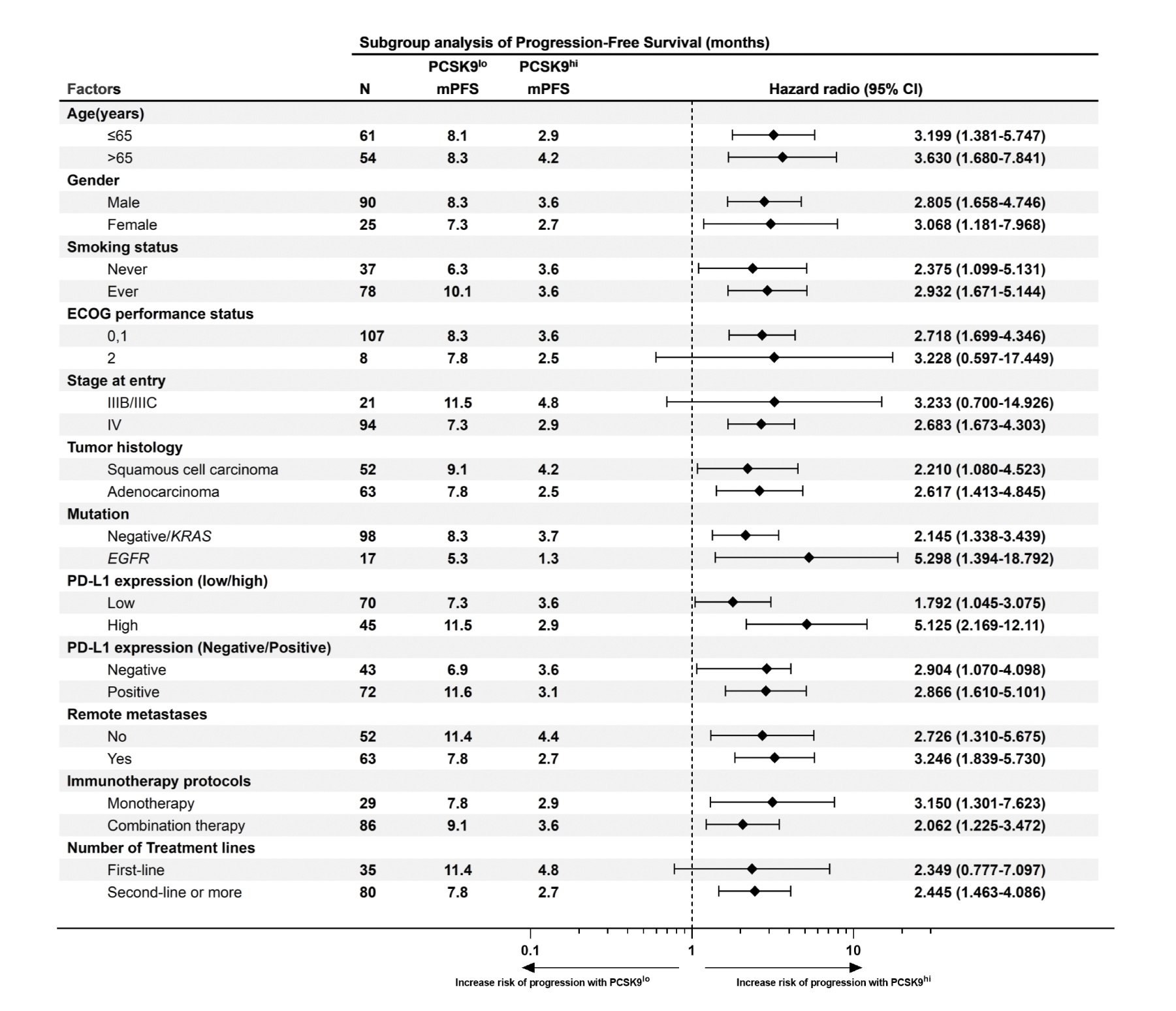

Supplement: Supplementary Figure 2 — Subgroup analysis for PFS of PCSK9hi and PCSK9lo group. *PD-L1 negative, TPS<1%; PD-L1 low, TPS<50%; PD-L1 high, TPS≥50%. [file Image_2.tif]

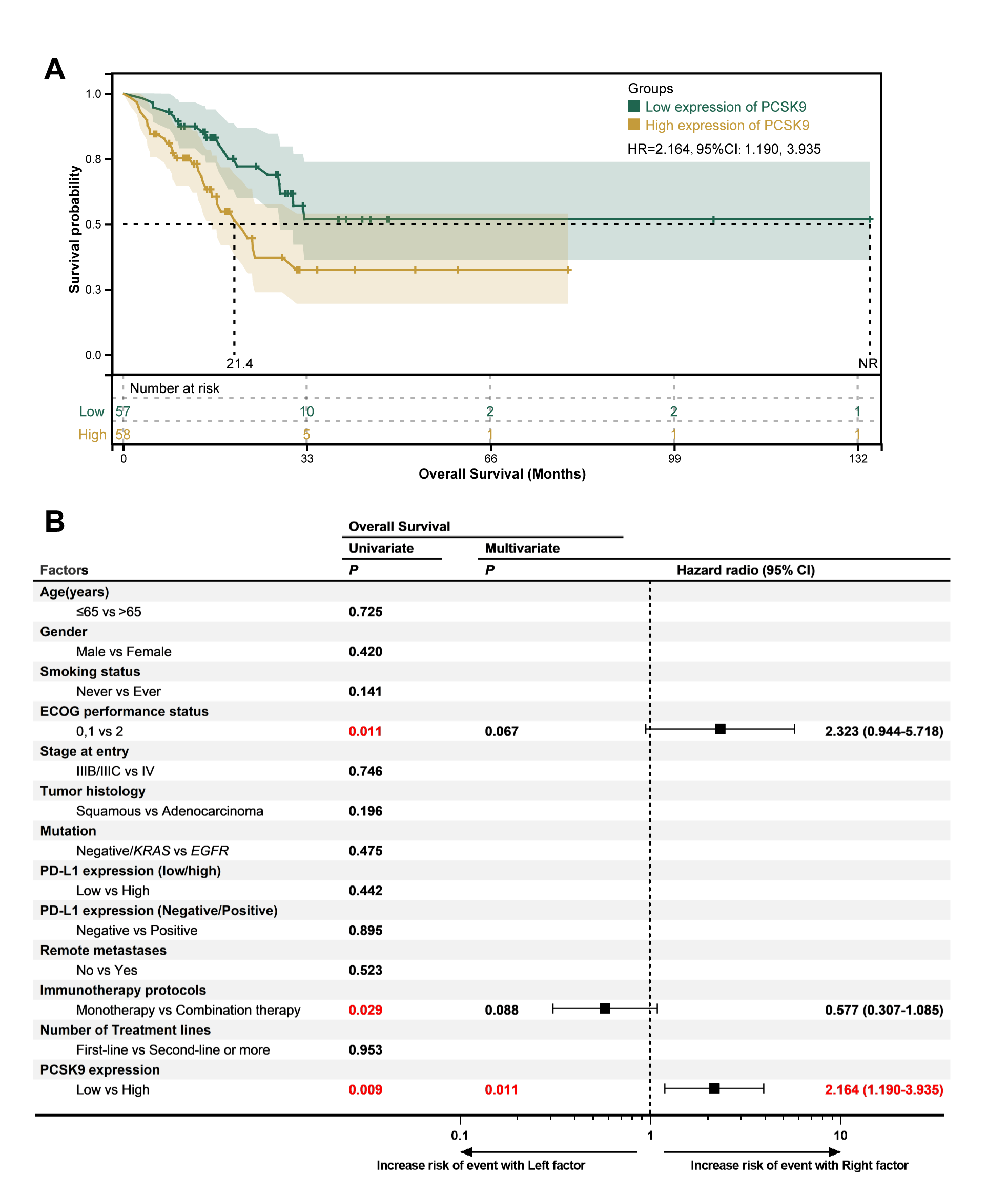

Supplement: Supplementary Figure 3 — PCSK9 was an independent risk factor for OS in advanced NSCLC patients with anti-PD1 immunotherapy. (A). Kaplan-Meier graph for overall survival in the population of PCSK9hi and PCSK9lo group. (B). Univariate and multivariate analyses of factors associated with overall survival. Data cutoff was April 30, 2022. *PD-L1 negative, TPS<1%; PD-L1 low, TPS<50%; PD-L1 high, TPS≥50%. [file Image_3.tif]
